# Correlation analysis of gamma-glutamyl transferase to lymphocyte ratio and patients with acute aortic syndrome in China: a propensity score-matched analysis

**DOI:** 10.3389/fcvm.2024.1333153

**Published:** 2024-07-01

**Authors:** Minhong Li, Weimin Xu, Hongchun Chen, Yidong Lai, Yequn Chen, Zhouwu Shu, Xuerui Tan

**Affiliations:** ^1^ Department of Cardiology, The First Affiliated Hospital of Shantou University Medical College, Shantou, China; ^2^ Department of Otolaryngology, Wuhan Fourth Hospital, Wuhan, China; ^3^ Shantou University Medical College, Shantou, China; ^4^ Clinical Research Center, The First Affiliated Hospital of Shantou University Medical College, Shantou, China

**Keywords:** acute aortic syndrome, gamma-glutamyl transferase to lymphocyte ratio, D-dimer, diagnosis, propensity score-matched analysis

## Abstract

**Background and objectives:**

Acute aortic syndrome (AAS) is a life-threatening condition in which there is a fracture in the integrity of the aortic wall. gamma-glutamyl transferase to lymphocyte ratio (GLR) is recognized as a risk factor for liver cirrhosis, fibrosis, and hepatocellular carcinoma. However, there are no clinical reports of GLR and AAS. We attempted to determine whether GLR level is associated with AAS in patients from the Chaoshan region of southern China.

**Methods:**

A total of 2,384 patients were recruited in this study and were divided into AAS and no-AAS groups according to the results of CT angiography of the thoracoabdominal aorta. Univariate and multivariate logistic regression was performed to identify risk factors for the occurrence of AAS. ROC was applied to assess the value of D-Dimer, GLR alone, or in combination for the diagnosis of AAS. And a 1:1 propensity score-matched analysis was performed.

**Results:**

Multivariate logistics regression analysis indicated that male, age, hypertension, diabetes, creatinine, D-dimer, and GLR were independent risk factors of AAS patients in the before propensity score-matching cohort. After propensity score-matching, it showed that D-dimer, GLR [OR 3.558(1.891, 6.697); *p* < 0.001] were independent risk factors of AAS patients. Before propensity score-matching, the area under the curve (AUC) was 0.822 of GLR and 0.767 of D-dimer. When both clinical backgrounds were adjusted, the AUC was 0.773 of GLR and 0.631 of D-dimer. GLR showed high specificity (80.5% and 77.1%), and D-dimer showed high sensitivity (84.7% and 73.6%) in the before and after propensity score-matching cohort.

**Conclusion:**

GLR and D-dimer were independent risk factors of acute aortic syndrome. D-dimer in combination with GLR is more valuable than a single indicator for diagnosing acute aortic syndrome.

## 1 Introduction

Acute aortic syndrome (AAS) is a life-threatening condition in which there is a fracture in the integrity of the aortic wall. The most common situations are aortic coarctation (AD), intramural hematoma (IMH), and penetrating atherosclerotic ulcer (PAU) (1, 2). AAS is characterized by non-specific signs and symptoms, complex causes, and easy misdiagnosis. Progression of the disease can lead to aortic dissection with a high mortality rate, and the risk increases with age. Therefore, timely and correct diagnosis is essential to increase patients’ chances of survival and prevent serious complications (3). Imaging is now the primary basis for confirming the diagnosis and classification of AAS (4), but there are not many biological markers that can guide clinical work. Nowadays the best imaging strategy is a combination of CT angiography and transthoracic echocardiography. Among biomarkers, D-dimer is the closest to “golden status” (high sensitivity and low negative likelihood ratio) (4).

Atherosclerosis (an inflammatory disease of the large arteries) is the leading candidate for cardiovascular disease. Therefore, the search for markers relating to atherosclerosis has implications for the prevention of AAS. Previous experiments have proven that serum gamma-glutamyl transferase (GGT) promotes the formation and development of atherosclerotic plaques, while lymphocytes are present in all stages of atherosclerosis (5). gamma-glutamyl transferase to lymphocyte ratio (GLR) is recognized as a risk factor for liver cirrhosis, fibrosis, and hepatocellular carcinoma (6). However, there are no clinical reports of GLR and AAS. We attempted to determine whether GLR level is associated with AAS in patients from the Chaoshan region of southern China.

## 2 Subjects and methods

### 2.1 Patient selection

**Figure 1:**
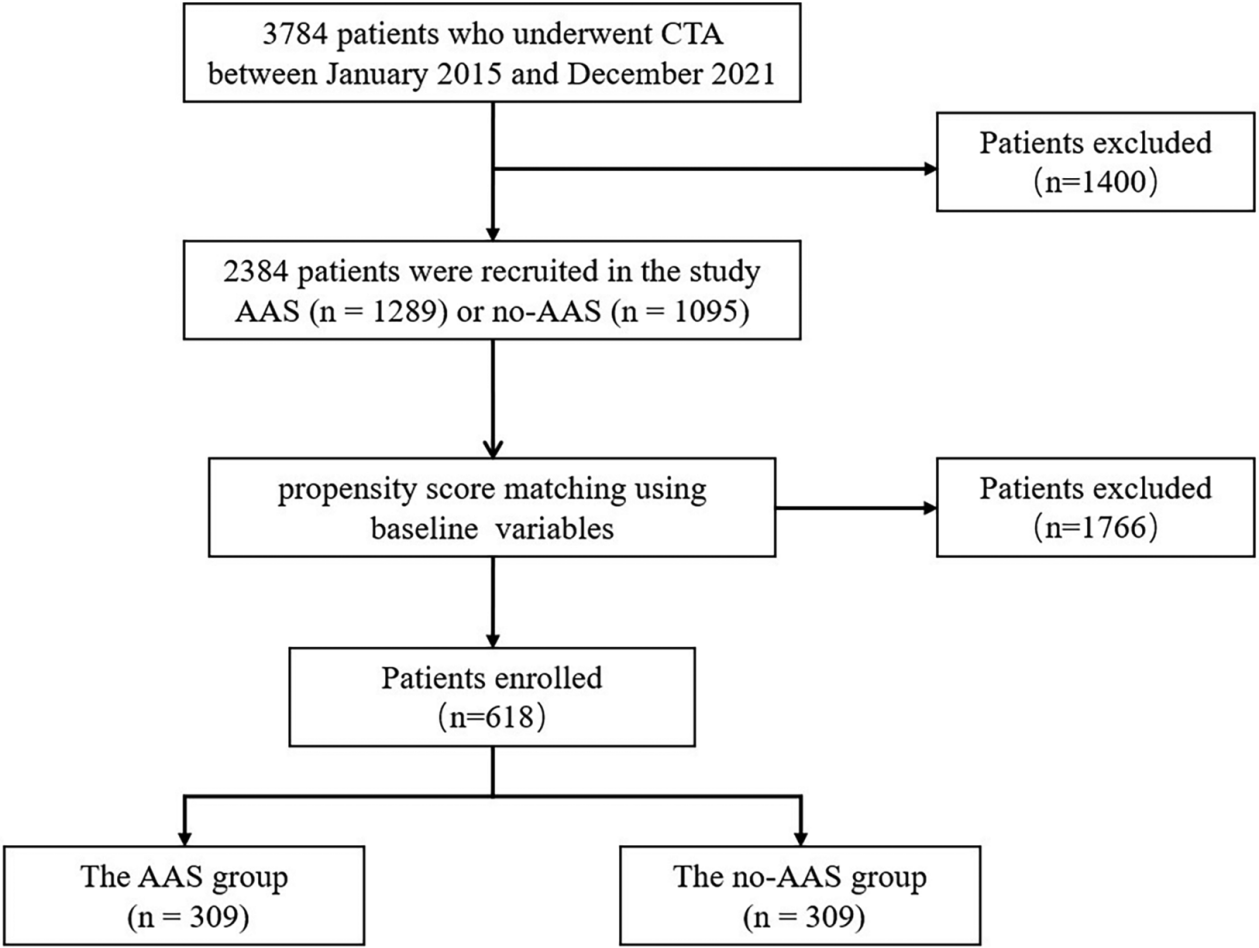
Grouping process.

In this study, 3,784 patients who underwent CT angiography of the thoracoabdominal aorta (CTA) at the First Affiliated Hospital of Shantou University Medical College between January 2015 and December 2021 were recruited. The exclusion criteria were as follows: ① congenital aortic anomalies, iatrogenic or traumatic aortic dissection, a history of cardiac or aortic surgery, pregnancy; ② patients with hematologic diseases, connective tissue diseases, biliary tract diseases, malignant tumors, chronic liver, and kidney diseases; ③patients on long-term use of glucocorticosteroids, NSAIDs, immunosuppressants and other drugs that affect the inflammatory response; ④non-first diagnosis with aortic dissection; ⑤ incomplete data. Ultimately, 2,384 patients were recruited in the study and divided into AAS and no-AAS cohorts based on CTA results (Figure 1).

### 2.2 Data collection and definitions

The clinical variables of enrolled patients were obtained through a review of medical records, which included age, gender, vital signs on admission (heart rate, systolic blood pressure, diastolic blood pressure), medical history (smoking, alcohol consumption, hypertension, diabetes, hyperlipidemia, CAD, and CVA). Laboratory data (aspartate aminotransferase, alanine aminotransferase, blood urea nitrogen, creatinine, uric acid, total cholesterol, triglycerides, high-density lipoprotein-cholesterol, low-density lipoprotein-cholesterol, gamma-glutamyl transferase, lymphocytes, D-dimer).

AAS is diagnosed by CTA, using the diagnostic criteria specified in *the 2022 ACC/AHA Guideline for the Diagnosis and Management of Aortic Disease* (7). The medical histories were based on the previous medical history documented in the hospital medical records. The venous blood samples were obtained within 24 h of admission at room temperature, and the report will be issued by the hospital laboratory physician. A propensity score-matched analysis is used to eliminate selection bias in studies by balancing the characteristics of participants between AAS and no-AAS cohorts.

### 2.3 Statistical analysis

All statistical analyses were processed using SPSS 26.0 and origin graphing, and a *p*-value of < 0.05 was considered statistically significant. Continuous variables were expressed as mean ± SD or median (interquartile range). All categorical data were presented as a percentage or an absolute number. The data were tested for normal distribution by the Kolmogorov–Smirnov test. Analyses of continuous variables were performed using independent *t*-test or Mann–Whitney *U* test and analyses of categorical variables were performed using chi-square test to assess differences between the two groups. The AAS cohorts were then 1-to-1 matched to the no-AAS cohorts on the propensity scores. The balancing covariates between the two groups include age, gender, vital signs on admission, medical history, and laboratory data (which is shown in Table 1). The procedure yielded 309 well-matched pairs. Between groups after propensity score-matching, the balance of measured variables was analyzed using a paired t-test for continuous measures and the McNemar test for categorical variables.

**Table 1:** Clinical characteristics of the study population.

	Before propensity matching			After propensity matching		
	AAS (*n* = 1,289)	No-AAS (*n* = 1,095)	*P*-value	AAS (*n* = 309)	No-AAS (*n* = 309)	*P*-value
Male (*n*, %)	985 (76.4%)	569 (52.0%)	<0.001	201 (65%)	208 (67.3%)	0.623
Age (years)	61.68 ± 12.33	59.47 ± 9.66	<0.001	62.39 ± 12.31	62.60 ± 9.68	0.811
Heart rate (beats/min)	79.61 ± 15.57	76.74 ± 12.49	<0.001	79.52 ± 15.82	78.02 ± 12.71	0.221
Systolic BP (mmHg)	156.22 ± 33.22	138.11 ± 40.88	<0.001	149.36 ± 28.06	148.20 ± 70.42	0.789
Diastolic BP (mmHg)	90.40 ± 20.31	83.31 ± 11.08	<0.001	86.79 ± 15.92	87.19 ± 12.20	0.717
Previous history (*n*, %)						
Smoking	566 (43.9%)	357 (32.6%)	<0.001	129 (41.7%)	120 (38.8%)	0.531
Alcohol consumption	185 (14.4%)	88 (8.1%)	<0.001	39 (12.6%)	34 (11.0%)	0.615
Hypertension	1,118 (86.7%)	548 (50.0%)	<0.001	239 (77.3%)	244 (79%)	0.699
Diabetes	158 (12.3%)	238 (21.8%)	<0.001	55 (17.8%)	63 (20.4%)	0.456
Hyperlipidemia	339 (26.3%)	323 (29.5%)	0.082	89 (28.8%)	85 (27.5%)	0.789
CAD	124 (9.6%)	73 (6.7%)	<0.001	32 (10.4%)	27 (8.7%)	0.583
CVA	98 (7.6%)	31 (2.8%)	<0.001	17 (5.5%)	18 (5.8%)	1.000
Laboratory variables						
AST (U/L)	22 (17, 35.11)	20.75 (17.36, 25)	<0.001	19.11 (16, 24)	21 (18, 25.78)	0.001
ALT (U/L)	18 (13, 30)	19 (14, 26)	0.944	16 (11.79, 23)	20 (15, 27)	<0.001
BUN (mmol/L)	6.95 (5.42, 9.14)	5.29 (4.45, 6.22)	<0.001	5.98 (4.84, 7.54)	5.43 (4.59, 6.37)	<0.001
Creatinine (umol/L)	105 (85.13, 133)	89 (77, 102)	<0.001	92 (77, 109)	97 (84.32, 111)	0.007
Uric acid (umol/L)	387.47 (309.22, 481.30)	370 (312.85, 436.55)	0.001	366 (283.49, 440.26)	393.60 (324.75, 448.10)	0.001
TC (mmol/L)	4.48 (3.75, 5.19)	4.93 (4.21, 5.65)	<0.001	4.57 (3.73, 5.37)	4.78 (4.02, 5.54)	0.002
TG (mmol/L)	1.16 (0.82, 1.66)	1.27 (0.93, 1.81)	<0.001	1.19 (0.82, 1.71)	1.33 (0.95, 1.73)	0.188
HDL-C (mmol/L)	1.10 (0.92, 1.30)	1.15 (0.96, 1.36)	<0.001	1.10 (0.90, 1.33)	1.10 (0.92, 1.28)	0.899
LDL-C (mmol/L)	2.85 (2.32, 3.42)	3.17 (2.66, 3.74)	<0.001	2.94 (2.32, 3.57)	3.07 (2.53, 3.65)	0.01
D-dimer (ug/LEU)	3,760 (1,705, 6,610)	690 (440, 2,390)	<0.001	2,570 (940, 4,910)	870 (520, 3,910)	0.02
GGT (U/L)	29.27 (20.75, 51)	25 (18, 35)	<0.001	26.93 (19.08, 44.84)	28 (20, 39)	<0.001
Lymphocytes L(*109 /L)	1.25 ± 0.71	2.03 ± 0.64	<0.001	1.33 ± 0.60	2.17 ± 0.71	<0.001
GLR	29.03 (16.60, 52.58)	13.06 (8.89, 18.91)	<0.001	21.35 (14.93, 37.69)	13.73 (9.39, 19.67)	<0.001

BP, blood pressure; CAD, coronary artery disease; CVA, cerebral vascular accident; AST, aspartate aminotransferase; ALT, alanine aminotransferase; BUN, blood urea nitrogen; TC, total cholesterol; TG, triglycerides; HDL-C, high-density lipoprotein-cholesterol; LDL-C, low-density lipoprotein-cholesterol; GGT, gamma-glutamyl transferase; GLR, gamma-glutamyl transferase to lymphocyte ratio.

Logistic regression analysis was used to explore the correlates affecting AAS, and the ratio was expressed as odds ratio (OR). ROC curves were used to assess the value of GLR, D-dimer, and the combination of both in the diagnosis of AAS.

## 3 Results

### 3.1 Patients’ characteristics

**Figure 2:**
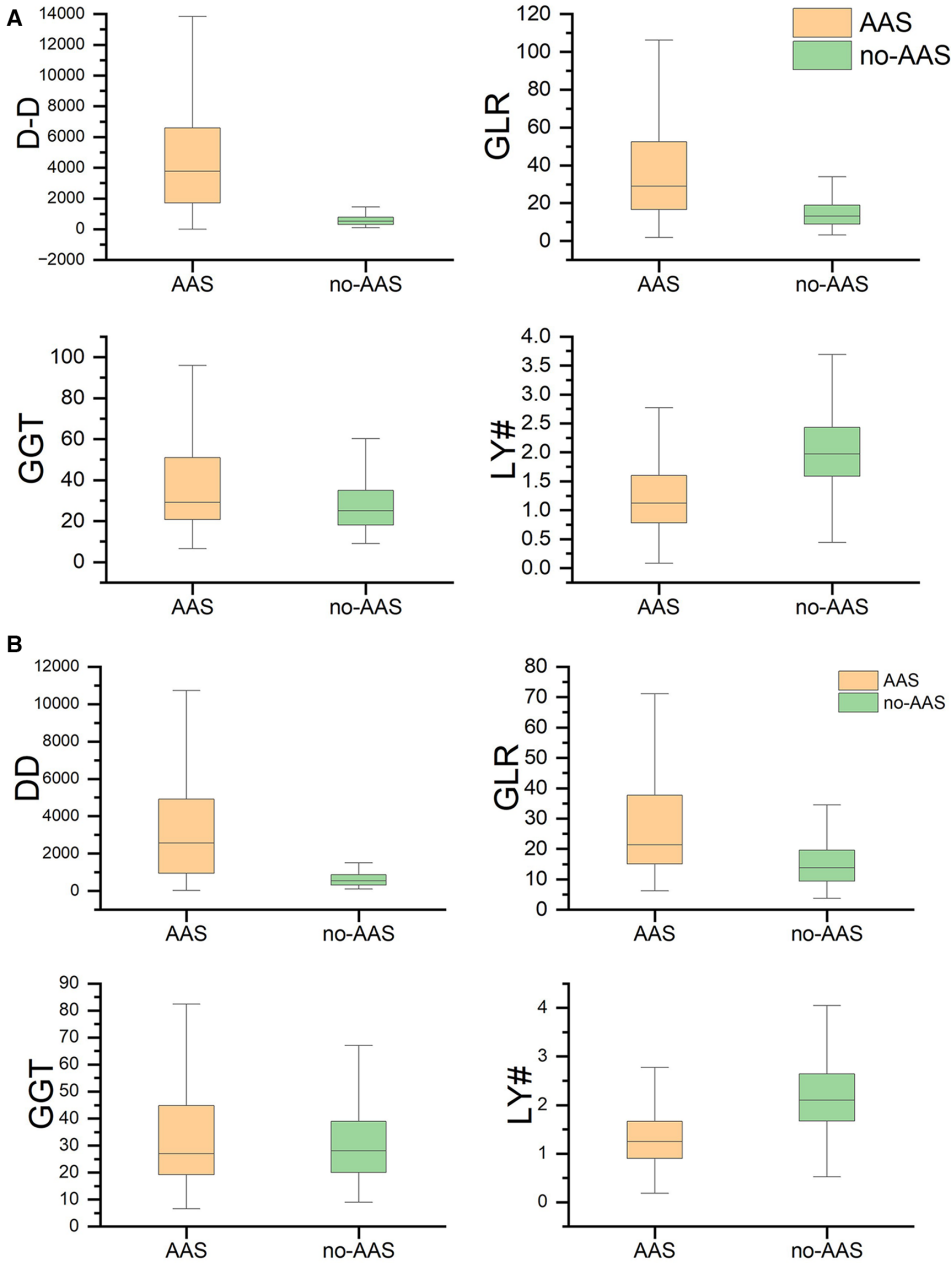
GGT, LY#, GLR, D-dimer levels between the no-AAS and AAS groups. (**A**) Before propensity matching cohort and (**B**) After propensity matching cohort. AAS, acute aortic syndrome; DD, D-dimer; GGT, gamma-glutamyl transferase; LY#, lymphocytes; GLR, gamma-glutamyl transferase to lymphocyte ratio.

A total of 2,384 patients were included in this study and were divided into AAS and no-AAS groups according to the results of CTA. The initial laboratory findings and baseline characteristics of the two groups are summarized in Table 1. It shows that the AAS and no-AAS groups had statistically significant variables except hyperlipidemia and ALT. To eliminate the uneven distribution of different factors between groups, propensity score matching was performed on both groups. When the covariates are balanced, a total of 309 matched pairs (309 patients from the AAS group and 309 patients from the no-AAS group) were generated. There were no significant differences in age, gender, vital signs on admission, or medical history, for the propensity score-matched subjects except for laboratory variables (Table 1). We can see that gamma-glutamyl transferase, D-dimer, and GLR were significantly higher in the AAS group than in the no-AAS group in both the before and after propensity score-matching cohorts. However, lymphocyte counts were higher in the no-AAS group (Table 1; Figures 2A,B).

### 3.2 Univariate analysis and multivariate logistics regression

Before propensity score-matching, multivariate logistics regression analysis indicated that male, age, hypertension, diabetes, creatinine, D-dimer, and GLR were independent risk factors of AAS patients. (Table 2A) After propensity score-matching, Univariate analysis indicated that there were three variables including total cholesterol(TC), D-dimer, and GLR with a *p*-value <0.05. All variables were put into forward conditional logistic regression analysis and found that D-dimer [OR 1.010 (1.003, 1.017); *p* = 0.002], GLR [OR 3.558(1.891, 6.697); *p* < 0.001] were independent risk factors of AAS patients (Table 2B).

**Table 2:** Univariate and multivariate logistics regression analysis for AAS and no-AAS groups.

(A) Before propensity matching cohort						
	Univariate			Multivariate		
	OR	95.0%CI	*P*-value	OR	95.0%CI	*P*-value
Male (*n*, %)	2.995	2.515–3.568	<0.001	0.312	0.180∼0.542	<0.001
Age (years)	1.018	1.010–1.025	<0.001	1.022	1.001∼1.044	0.043
Heart rate (beats/min)	1.014	1.009–1.020	<0.001	0.995	0.980∼1.010	0.478
Smoking (*n*, %)	1.618	1.369–1.913	<0.001	1.468	0.867∼2.485	0.153
Alcohol consumption (*n*, %)	1.914	1.464–2.501	<0.001	0.881	0.421∼1.844	0.737
Hypertension (*n*, %)	6.526	5.344–7.970	<0.001	0.149	0.085∼0.260	<0.001
Diabetes (*n*, %)	0.502	0.403–0.625	<0.001	0.345	0.193∼0.619	<0.001
Hyperlipidemia (*n*, %)	1.068	0.896–1.273	0.465			
AST (U/L)	1.034	1.026–1.041	<0.001	1.015	0.995∼1.036	0.145
ALT (U/L)	1.012	1.008–1.017	<0.001	0.992	0.980∼1.005	0.224
BUN (mmol/L)	1.055	1.027–1.083	<0.001	0.997	0.983∼1.011	0.675
Creatinine (u mol/L)	1.014	1.012–1.017	<0.001	1.037	1.025∼1.050	<0.001
Uric acid (u mol/L)	1.001	1.000–1.001	<0.001	1.001	1.000∼1.002	0.191
TC (mmol/L)	1.000	0.993–1.007	0.999			
LDL-C (mmol/L)	0.931	0.852–1.017	<0.001	0.922	0.839∼1.012	0.087
D-dimer (ug/LFEU)	1.024	1.019–1.028	<0.001	1.019	1.007∼1.038	0.043
GLR	1.094	1.083–1.104	<0.001	1.030	1.013∼1.046	<0.001
(B) After propensity matching cohort						
	Univariate			Multivariate		
	OR	95.0%CI	*P*-value	OR	95.0%CI	*P*-value
Male (*n*, %)	1.107	0.793–1.544	0.552			
Age (years)	0.998	0.984–1.013	0.810			
Heart rate (beats/min)	1.007	0.996–1.019	0.197			
Smoking (*n*, %)	1.129	0.818–1.557	0.461			
Alcohol consumption (*n*, %)	0.856	0.525–1.397	0.533			
Hypertension (n,%)	1.099	0.750–1.611	0.626			
Diabetes (*n*, %)	1.183	0.791–1.768	0.413			
Hyperlipidemia (*n*, %)	1.066	0.751–1.514	0.721			
AST (U/L)	0.998	0.984–1.012	0.759			
ALT (U/L)	0.991	0.981–1.002	0.127			
BUN (mmol/L)	0.998	0.988–1.009	0.752			
creatinine (u mol/L)	1.000	0.998–1.002	0.941			
uric acid (u mol/L)	1.000	0.999–1.000	0.769			
TC (mmol/L)	0.799	0.706–0.905	<0.001	0.795	0.296∼2.14	0.650
LDL-C (mmol/L)	1.008	0.967–1.051	0.716			
D-dimer (ug/LFEU)	1.000	1.000–1.001	0.003	1.010	1.003∼1.017	0.002
GLR	1.074	1.055–1.094	<0.001	3.558	1.891∼6.697	<0.001

OR, odds ratio; CI, confidence interval; AST, aspartate aminotransferase; ALT, alanine aminotransferase; BUN, blood urea nitrogen; TC, total cholesterol; LDL-C, low-density lipoprotein-cholesterol; GLR, gamma-glutamyl transferase to lymphocyte ratio.

(A) Before propensity matching cohort and (B) after propensity matching cohort.

### 3.3 The receiver operating characteristic (ROC) analysis

**Figure 3:**
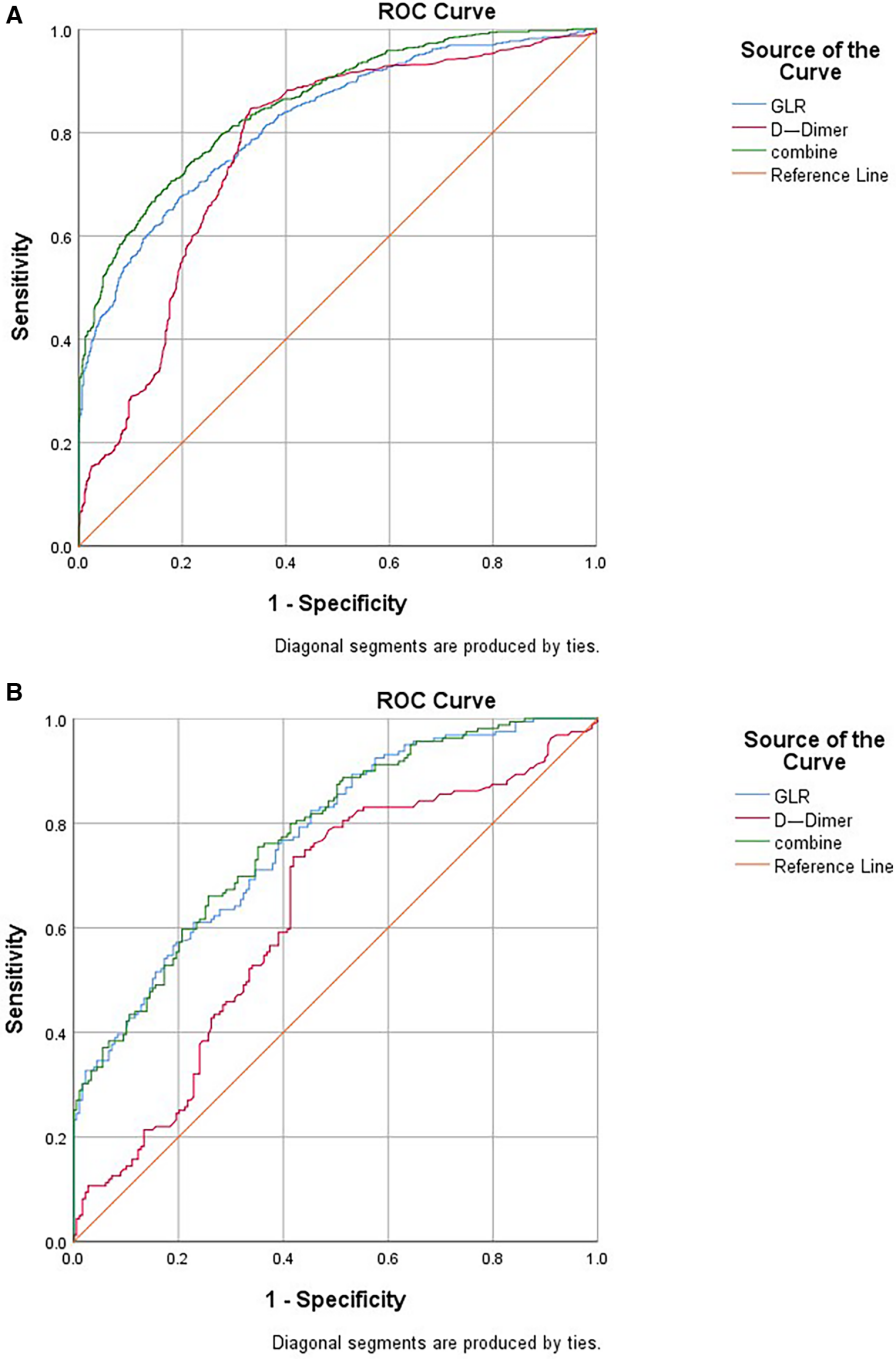
ROC curve indicating the predictive value of GLR and D-dimer. (**A**) Before propensity matching cohort and (**B**) After propensity matching cohort. ROC, receiver operating characteristic; GLR, gamma-glutamyl transferase to lymphocyte ratio.

According to logistic regression analysis, GLR and D-dimer were independent risk factors of the AAS group in both the before and after propensity score-matching cohort. Therefore, GLR and D-dimer are included in ROC, while the joint index of GLR and D-dimer is obtained using logistic regression analysis. Before propensity score-matching, the area under the curve (AUC) was 0.822 of GLR and 0.767 of D-dimer. When both clinical backgrounds were adjusted, the AUC was 0.773 of GLR and 0.631 of D-dimer. GLR showed high specificity (80.5% and 77.1%), and D-dimer showed high sensitivity (84.7% and 73.6%) in the before and after propensity score-matching cohort (Figure 3; Table 3).

**Table 3:** ROC curve indicating the predictive value of GLR and D-dimer.

	Before propensity matching			After propensity matching		
	D-dimer	GLR	GLR + D-dimer	D-dimer	GLR	GLR + D-dimer
Cut-off value	995	20.264	0.466	1,025	19.123	0.367
Sensitivity	0.847	0.675	0.706	0.736	0.61	0.755
Specificity	0.667	0.805	0.822	0.581	0.771	0.648
Youden's index	0.514	0.48	0.528	0.317	0.381	0.403
+LR	2.544	3.462	3.966	1.757	2.664	2.145
−LR	0.229	0.404	0.358	0.454	0.506	0.378
AUC	0.767	0.822	0.854	0.631	0.773	0.778
95%CI	0.743–0.792	0.801–0.842	0.836–0.872	0.571–0.690	0.724–0.821	0.730–0.826
*P*-value	<0.001	<0.001	<0.001	<0.001	<0.001	<0.001

AUC, the area under receiver-operator characteristic; CI, confidence interval; GLR, gamma-glutamyl transferase to lymphocyte ratio; −LR, negative likelihood ratio; +LR, positive likelihood ratio.

## 4 Discussion

Recent evidence (8) suggests that inflammation is causally associated with different clinical manifestations of aortic disease. It is composed of acute aortic syndromes (AD, IMH, and PAU), occlusive aortic disease, vasculitis of the aortic wall (Takayasu arteritis, Reactive Arthritis as well as others), and so on. However, our study is primarily concerned with AAS. In this research, GLR and D-dimer are independent risk factors of patients with AAS. GLR + D-dimer provided additive predictive value over either marker alone. The finding may be beneficial to future clinical work.

AAS is a group of aortic lesions with similar clinical symptoms but different pathological mechanisms (8). Whether due to inherent instability of the aortic wall (e.g., in the case of hereditary connective tissue disease) or acquired factors (e.g., atherosclerotic degeneration due to aging) (9), disruption of its structural integrity is a key prerequisite for the development of aortic disease. Thus, the study was conducted with the exclusion of AAS due to inherent instability as much as possible. Endothelial rupture or vascular ulceration leads to blood flow into the wall or rupture of the trophoblastic vessels supplying the aortic wall, both of which can cause damage to the aortic wall and thus induce pathological changes in the aorta (10, 11). Aortic wall degeneration is associated with aging, atherosclerosis, hypertension, and accompanying inflammation. Extracellular matrix degradation and inflammation production are currently considered to be the main mechanisms leading to arterial wall damage (8).

In recent decades, circulating biomarkers with an established role in the diagnostic-prognostic pathway of AAS. Among these, D-dimer appears to be the closest to acquiring “golden status” (12, 13). In clinical work, D-dimer, neutrophil to lymphocyte ratio(NLR) (14), and C-reaction protein(CRP) (15, 16) predictors were the most commonly used biomarkers in patients with AAD, the performance of prognostic factors showed a poor to strong discrimination (17). Exploring new biological markers may provide better coverage for patients. D-dimer testing is widely used in acute aortic dissection (AAD) as a diagnostic-prognostic marker in the clinical arena, A cutoff level of 0.5 mg/L can reliably be used to rule out AAD (4). According to logistic regression analysis in this study, GLR and D-dimer were independent risk factors of the AAS group in both the before and after propensity score-matching cohort. The ROC showed that the area under the curve of D-dimer, GLR, and GLR + D-dimer was between 0.6 and 1.0. It suggests that three variables have certain clinical value in the diagnosis of AAS. In clinical practice, CTA should be performed more actively in patients with significantly elevated levels of GLR and D-dimer.

Lymphocytes infiltrate the aortic wall and cause apoptosis of smooth muscle cells through the Fas-Fasl pathway, which further leads to impaired vascular cell structure, disruption of matrix homeostasis, and thinning of the arterial wall, which in turn induces lesions (18, 19). CD + 8 cells have direct cytotoxic effects and also promote cytokine secretion, which leads to vascular remodeling and rupture. Previously, flow cytometry was used (20) to analyze various lymphocyte subsets in patients with acute aortic coarctation. The results showed that AAD exhibited a decrease in CD4^+^ T and CD8^+^ *T* cells and that low CD4^+^ T lymphocyte count was an independent risk factor for poor postoperative prognosis. That's why Table 1 shows that lymphocytes in the AAS group were significantly less than the no-AAS group [1.25 ± 0.71 vs. 2.03 ± 0.64, *p* < 0.05]. Sebastiano Cicco et al. (21) found CD3+ CD4 + cell infiltrates were detected in the vessel wall samples of Takayasu arteritis patients(excluded from our study), whose mean proportion of Tregs was smaller than Controls(age- and sex-matched atherosclerotic patients)at T0, but increased significantly at T18. It needs to be followed up with further studies.

Elevated serum GGT levels are an independent indicator of activation of systemic inflammatory responses and enhanced oxidative stress, which is of widespread significance in atherosclerosis formation. Serum GGT concentrations are significantly associated with ascending aortic dilation (22). It has also been reported to be associated with arterial stiffness, impaired aortic elasticity, and blood pressure (23). Therefore, it is reasonable to believe that there is a correlation between elevated serum GGT levels and the occurrence of AAS. Based on the above mechanisms, We believe that GLR plays an influential role in the development and progression of aortic atherosclerosis.

The majority of variables were statistically significant between the AAS and no-AAS groups. To eliminate the uneven distribution of different factors between groups, propensity score matching was performed on both groups. Let the variables between the groups be balanced as much as possible to reliably justify whether there is a correlation between GLR and AAS. According to logistic regression analysis, D-dimer [OR 1.010 (1.003, 1.017); *p* = 0.002], GLR [OR 3.558 (1.891, 6.697); *p* < 0.001] were independent risk factors of the AAS group. The ROC showed that the sensitivity of D-dimer for diagnosing AAS was high, while the specificity of GLR was better, and the area under the curve of GLR combined with DD for diagnosing AAS was higher than that of a single indicator. It indicates that the two indicators can complement each other and may be able to assist with the diagnosis of AAS. An international multicenter prospective study by Dr. Nazerian et al. (24) from Italy showed that the overall sensitivity of a positive D-dimer level for the diagnosis of AAS was 96.7% and the specificity was 64%. Consistent with the results of this study. *2014 ESC Guidelines on the diagnosis and treatment of aortic diseases* (25) have included D-dimer testing in the AAS diagnostic workup.

### 4.1 Limitations

This study has several limitations. First, the source of our patients is only from the Chaoshan area, which may have biased the analysis results. Secondly, this study was performed in a group of aortic diseases (AAS) without an in-depth exploration of aortic dissection, intramural hematoma, and penetrating atherosclerotic ulcer et al.

### 4.2 Future directions

In summary, GLR and D-dimer were independent risk factors of acute aortic syndrome. D-dimer in combination with GLR is more valuable than a single indicator for diagnosing AAS. Therefore, CTA should be performed more aggressively in patients with significantly elevated GLR and D-dimer levels in clinical work. By doing this it may be possible to reduce the rate of misdiagnosis and avoid the waste of medical resources. In the next few moments, it is hoped that relevant experiments were designed to elucidate the potential mechanism of GLR and AAS and to explore the rationality of its application in clinical settings.

## 5 Conclusion

GLR and D-dimer were independent risk factors of acute aortic syndrome. D-dimer in combination with GLR is more valuable than a single indicator for diagnosing acute aortic syndrome.

## Data Availability

The raw data supporting the conclusions of this article will be made available by the authors, without undue reservation.
